# Lethal and Sublethal Toxicity Assessment of Cyclosporin C (a Fungal Toxin) against *Plutella xylostella* (L.)

**DOI:** 10.3390/toxins14080514

**Published:** 2022-07-28

**Authors:** Jianhui Wu, Xiaochen Zhang, Muhammad Hamid Bashir, Shaukat Ali

**Affiliations:** 1 Key Laboratory of Bio-Pesticide Innovation and Application, College of Plant Protection, South China Agricultural University, Guangzhou 510642, China; jhw@scau.edu.cn (J.W.); zhangxiaoying@stu.scau.edu.cn (X.Z.); 2 Engineering Research Center of Biological Control, Ministry of Education and Guangdong Province, South China Agricultural University, Guangzhou 510642, China; 3 Department of Entomology, Faculty of Agriculture, University of Agriculture, Faisalabad 38000, Pakistan; hamid_uaf@yahoo.com

**Keywords:** biopesticides, cyclosporin C, diamondback moth, toxicity, antifeedant activity, fecundity, enzyme activities

## Abstract

Secondary metabolites/toxins produced by *Purpeocillium lilacinum* (Hypocreales; Phiocordycipitaceae), a well-known insect pathogen, can be used for the management of different insect pests. We report the lethal and sublethal effects of cyclosporin C (a toxin produced by *P. lilacinum*) against a major vegetable pest, *Plutella xylostella*, at specific organismal (feeding rate, larval growth, adult emergence, fecundity, and adult longevity) and sub-organismal levels (changes in antioxidant and neurophysiological enzyme activities). The toxicity of cyclosporin C against different larval instars of *P. xylostella* increased with increasing concentrations of the toxin and the maximum percent mortality rates for different *P. xylostella* larval instars at different times were observed for the 300 µg/mL cyclosporin C treatment, with an average mortality rate of 100% for all larval instars. The median lethal concentrations (LC_50_) of cyclosporin C against the first, second, third, and fourth larval instars of *P. xylostella* 72 h post-treatment were 78.05, 60.42, 50.83, and 83.05 μg/mL, respectively. Different concentrations of cyclosporin C caused a reduction in the average leaf consumption and average larval weight. Different life history parameters, such as the pupation rate (%), adult emergence (%), female fecundity, and female longevity were also inhibited when different concentrations of cyclosporin C were applied topically. The cyclosporin C concentrations inhibited the activities of different detoxifying (glutathione S-transferase, carboxylesterase, and acetylcholinesterase) and antioxidant enzyme (superoxide dismutase, catalase, and peroxidase) activities of *P. xylostella* when compared to the control. These findings can serve as baseline information for the development of cyclosporin C as an insect control agent, although further work on mass production, formulation, and field application is still required.

## 1. Introduction

Different species of insect pathogenic fungi are possible alternatives to chemical insecticides for insect pest management. Their diversity (among taxa), production of secondary metabolites, and environmental safety make them more practically applicable biological control agents [1]. Members of fungal taxa, *Purpureocillium* Luangsa-ard, are pathogens of plant-feeding insects and nematodes [2,3]. *Purpureocillium lilacinum* is a well-known insect pathogen that has been used to control different crop pests, such as aphids, whiteflies, thrips, fruit flies, and plant parasitic nematodes [2,3,4]. *Purpureocillium lilacinum* isolates produce different secondary compounds/toxins known as paecilotoxins [5]. These toxins demonstrate oral toxicity against insects and nematodes [6].

Cyclosporins are nonpolar cyclic oligopeptides that are produced by different fungal species belonging to genera such as *Beauveria*, *Lecanicillum*, *Purpeocillium*, and *Tolypocladium* [5,7]. During insect infection, cyclosporins are secreted at later stages of host infection and disrupt the immune response of the insect host, ultimately leading to insect mortality [8,9,10]. Cyclosporins act through the blocked glycoprotein-related pumps involved in the removal of xenobiotics out of haemolymph [11]. Weiser and Matha [12] reported the toxicity mechanism of cyclosporin A against *Culex pipiens.* Cyclosporin A toxicity induced different histopathological changes to *C. pipiens* tissues (formation of swollen mitochondria and vacuolization of the endoplasmic reticulum) [13]. Vilcinskas et al. [5] reported that an artificial diet supplemented with a sublethal concentration of cyclosporin A reduced the phagocytosis of plasmatocytes in *Galleria mellonella*. Mosquito (*Aedes aegypti*) larvae treated with dried methanolic extract of *Tolypocladium* (with cyclosporin A as the predominant metabolite) caused high mortality because of the disruption of midgut mitochondrial cells [14]. Cyclosporins have been reported to show a narrow spectrum of antibiotic and immuno-suppressive activities in humans, recently being used for the cure of different diseases [15,16,17,18,19], but the use of cyclosporins at high doses (above 25 mg/kg) can cause adverse effects, including nephrotoxicity, diarrhoea, headache, anxiety, and metabolic toxicity [20,21]. However, the use of cyclosporins at lower doses (below 3 mg/kg) was reported to have no side effects [22]. Recent studies have shown that the immuno-suppressive effects of cyclosporin C are lower than that of other cyclosporins [15], and therefore, it would be of interest to study methods of mass production and the insecticidal efficacy of cyclosporin C produced by insect pathogenic fungi *P. lilacinum*, keeping in mind the ecological implications of its application. Since cyclosporin C has been reported to be produced by different plant pathogenic filamentous fungi (belonging to the genera *Aspergillus* and *Fusarium*) [23], cyclosporin C should be used at low doses (below 3 mg/kg) for insect pest management to avoid the risks of mycotoxin contamination and indirectly to avoid public health risks from the consumption of mycotoxin-contaminated foods [24].

*Plutella xylostella* (L.) (Lepidoptera: Plutellidae) is a major threat to different vegetable crops across the globe. The annual yield losses caused by *P. xylostella* worldwide are estimated to be USD 4–5 billion [25]. This insect pest has a strong ability to develop insecticide resistance [26]. Therefore, the use of insect pathogenic fungi or their secondary metabolites can serve as an alternate strategy for *P. xylostella* management. Amiri et al. [27] showed a reduction in the feeding rate of *P. xylostella* in response to destruxin (a fungal toxin produced by *Metarhizium anisopliae*) application. The observed antifeedant index (AI) was dose-related, and there were significant differences between the treated and untreated leaves. Huang et al. [28] observed increased mortality of *P. xylostella* larvae with increasing concentrations of destruxin A.

So far, very minimal work has been reported on the lethal and sublethal effects of cyclosporin C on target insect hosts [29]. The sublethal effects have been observed on a life history basis (fecundity/life table parameters) [28] and an immune response basis (variations in activities of antioxidant and neurophysiological enzymes) [30]. The present work was performed to assess the lethal and sublethal toxicity of cyclosporin C against *P. xylostella*. The lethal toxicity was studied by testing the efficacy of different cyclosporin C concentrations against different larval instars of *P. xylostella*. The sublethal toxicity was observed at specific organismal (feeding rate, change in larval weight, adult emergence, fecundity, and adult longevity) and sub-organismal levels (changes in antioxidant and neurophysiological enzyme activities). We hope that this study will provide the basis for the future development of cyclosporin C as a biological control agent for the management of insect pests such as *P. xylostella.*

## 2. Results

### 2.1. Toxicity of Cyclosporin C against P. xylostella

#### 2.1.1. Concentration–Mortality Response of *P. xylostella* to Cyclosporin C

**Figure 1:**
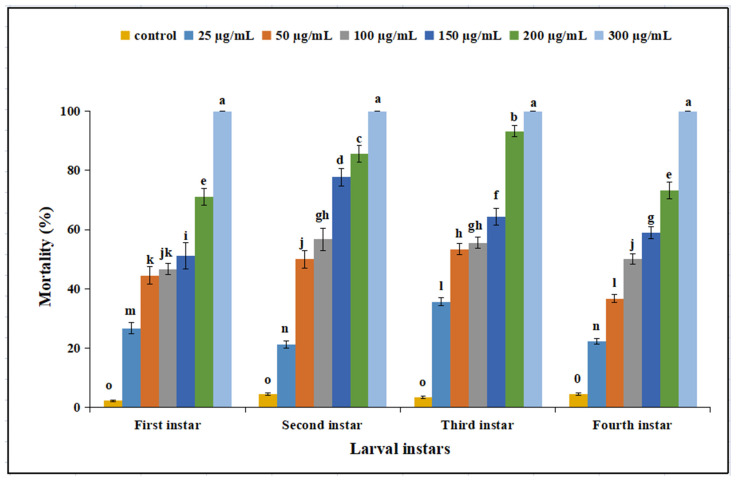
Mortality (%) of different larval instars of *Plutella xylostella* treated with different concentrations of cyclosporin C. Bars represent standard error of means (based on three independent replicates). Bars with different letters represent significantly different means (Tukey’s, *p* < 0.05).

After 3 days of treatment, the percent mortality rates of different *P. xylostella* larval instars were significantly different among the different treatments and the control (F_18,56_ = 6 6.45; *p* < 0.01). The mortality of *P. xylostella* increased with an increasing concentration of cyclosporin C (Figure 1). The maximum percent mortality rates for different *P. xylostella* larval instars at different times were observed for the 300 µg/mL cyclosporin C treatment, with an average mortality rate of 100% for all larval instars.

#### 2.1.2. Transmission Electron Microscopic Examination of *P. xylostella* Midgut following Cyclosporin C Treatment

**Figure 2:**
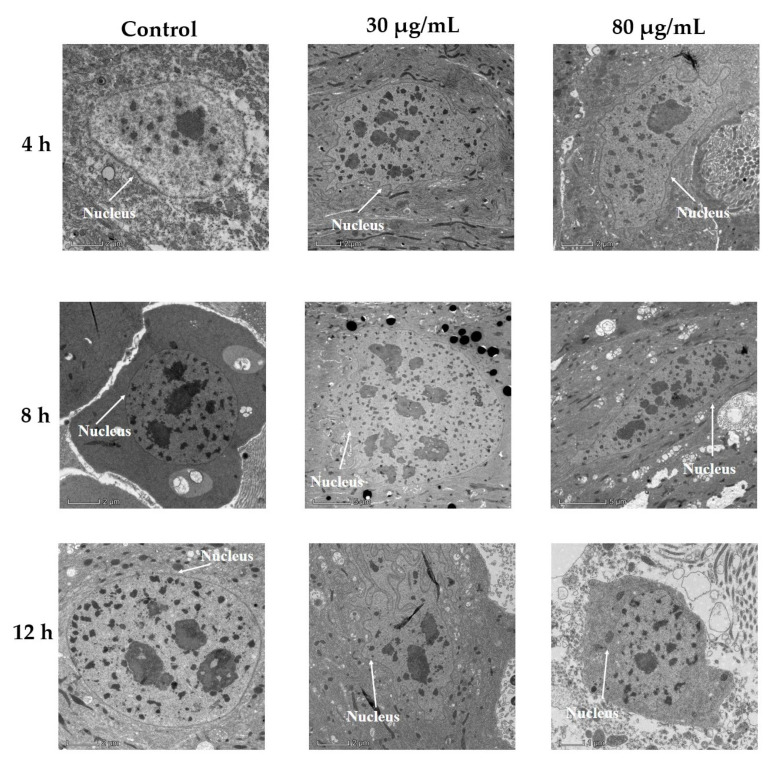
Changes in nucleus region of fourth *Plutella xylostella* (fourth instar larvae) midgut cells following cyclosporin C treatment.

As shown in Figure 2, the nuclei of untreated cells had smooth nuclear membranes, but after ingesting cyclosporin C, the nuclei of midgut cells were enlarged, and the nuclear membranes appeared to be folded. The degree of nuclei enlargement increased, and the nuclear membrane folds became more obvious with an increase in time post-treatment. However, the nuclei and nuclear membranes of the untreated midgut cells did not change much.

**Figure 3:**
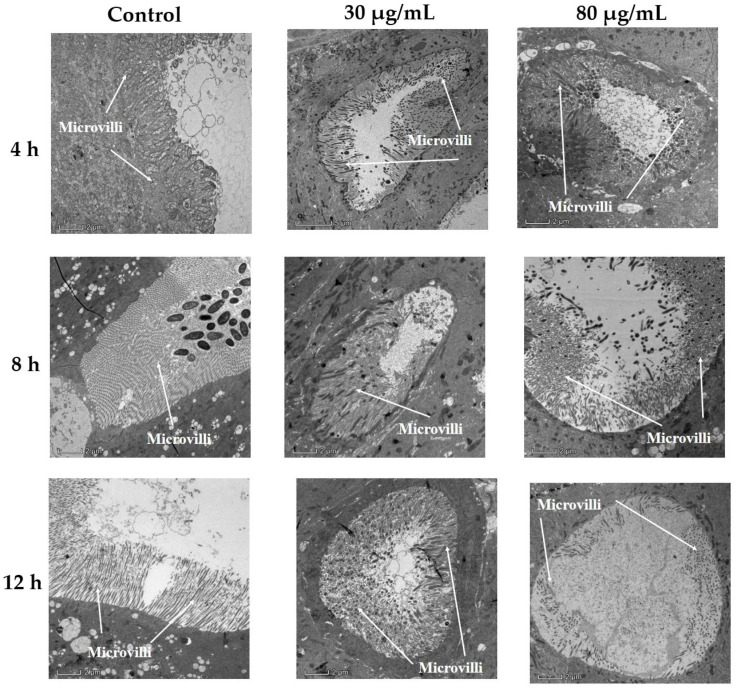
Changes in microvilli of *Plutella xylostella* (fourth instar larvae) midgut cells following cyclosporin C treatment.

The microvilli of treated cells became shorter and more disorganized with the passage of time (Figure 3). The microvilli of the midgut cells from *P. xylostella* larvae treated with an 80 µg/mL concentration became completely detached and minute in size after 12 h of treatment when compared to the control cells (Figure 3).

### 2.2. Sublethal Effects of Cyclosporin C against P. xylostella

#### 2.2.1. Effect of Cyclosporin C on Feeding and Larval Weight of *P. xylostella*

**Figure 4:**
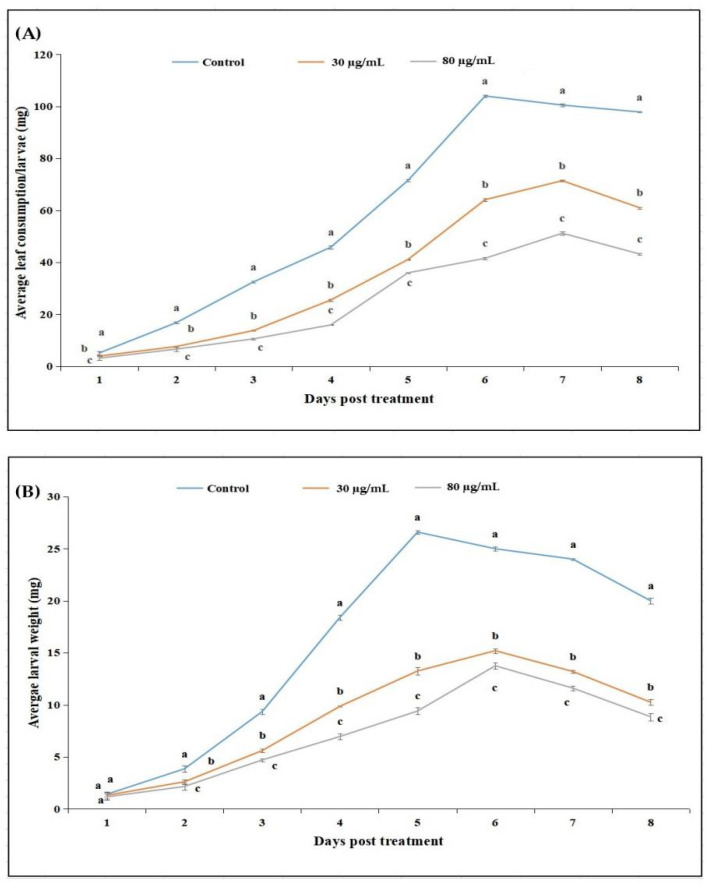
Amount of cabbage leaf consumed (**A**) and change in larval body weight (**B**) of *Plutella xylostella* larvae treated with cyclosporin C at different time intervals. Lines represent standard error of means (based on three independent replicates). Lines with different letters represent significantly different means (Tukey’s, *p* < 0.05).

The average feeding rates (milligrams of leaves consumed per larvae) of *P. xylostella* larvae at different time intervals were significantly different among the cyclosporin C treatments and the control (F_14,68_ = 26.92, *p* < 0.0001). A decrease in the amount of leaf consumed (mg) by *P. xylostella* larvae was observed with increases in the cyclosporin C concentration. The amount of leaf consumed (mg) by *P. xylostella* larvae treated with the 30 μg/mL cyclosporin C treatment across the experimental period was significantly higher than that of those treated with the 80 μg/mL cyclosporin C treatment, but it was still significantly lower than that of the control (Figure 4A).

The average weight (mg) of *P. xylostella* larvae at different time intervals were significantly different among the cyclosporin C treatments and the control (F_14,68_ = 26.92, *p* < 0.0001). A decrease in the average weight (mg) of *P. xylostella* larvae was observed with increases in the cyclosporin C concentration. The average weight (mg) of *P. xylostella* larvae treated with 80 μg/mL cyclosporin C was significantly lower than the larval weights observed for those under the 30 μg/mL cyclosporin C treatment (except one day post-treatment) (Figure 4B).

#### 2.2.2. Sublethal Toxicity of Cyclosporin C

The pupation rate (%) of *P. xylostella* after 10 days of treatment was significantly different among the different cyclosporin C treatments and the control (F_2,5_ = 21.69, *p* < 0.001). Maximum pupation (96.66 ± 1.66%) was observed for the control, whereas the rates of pupation observed for the 30 μg/mL and 80 μg/mL cyclosporin C treatments were 58.33 ± 1.40 and 14.44 ± 0.93%, respectively (Table 1).

The adult emergence (%) of *P. xylostella* after 10 days of treatment was significantly different among the different cyclosporin C treatments and the control (F_2,5_ = 34.84, *p* < 0.001). The maximum adult emergence (66.66 ± 2.33%) was observed for the control, whereas the rates of adult emergence observed for the 30 μg/mL and 80 μg/mL cyclosporin C treatments were 23.33± 1.66 and 7.78 ± 1.11%, respectively (Table 1).

The pre-oviposition periods of *P. xylostella* after 10 days of treatment were significantly different among the different cyclosporin C treatments and the control (F_2,5_ = 36.97, *p* < 0.001). The longest pre-oviposition period (11.50 ± 0.33 hrs) was observed for 80 μg/mL cyclosporin C, whereas the pre-oviposition periods of *P. xylostella* observed for the 30 μg/mL treatment and the control were 9.33 ± 0.50 and 7.25 ± 0.66 hrs, respectively (Table 1).

The fecundity of *P. xylostella* adults that emerged from fourth instar larvae treated with different cyclosporin C concentrations was significantly different among different treatments and the control (F_2,5_ = 43.26, *p* < 0.001). The maximum fecundity (197 ± 4.48 eggs/female) was observed for the control, whereas the *P. xylostella* female fecundity observed for the 30 μg/mL and 80 μg/mL cyclosporin C treatments were 137 ± 2.93 and 82 ± 2.34 eggs/female, respectively (Table 1).

The longevity of *P. xylostella* adults that emerged from fourth instar larvae treated with different cyclosporin C concentrations was significantly different among different treatments and the control (F_2,5_ = 32.97, *p* < 0.001). The maximum *P. xylostella* female longevity (12 ± 0.33 days) was observed for the control, whereas the *P. xylostella* female longevity observed for the 30 μg/mL and 80 μg/mL cyclosporin C treatments were 9 ± 0.66 and 7 ± 0.66 days, respectively (Table 1).

#### 2.2.3. Effect of Cyclosporin C Treatment on Neurophysiological and Antioxidant Enzyme Activities of *P. xylostella*

**Figure 5:**
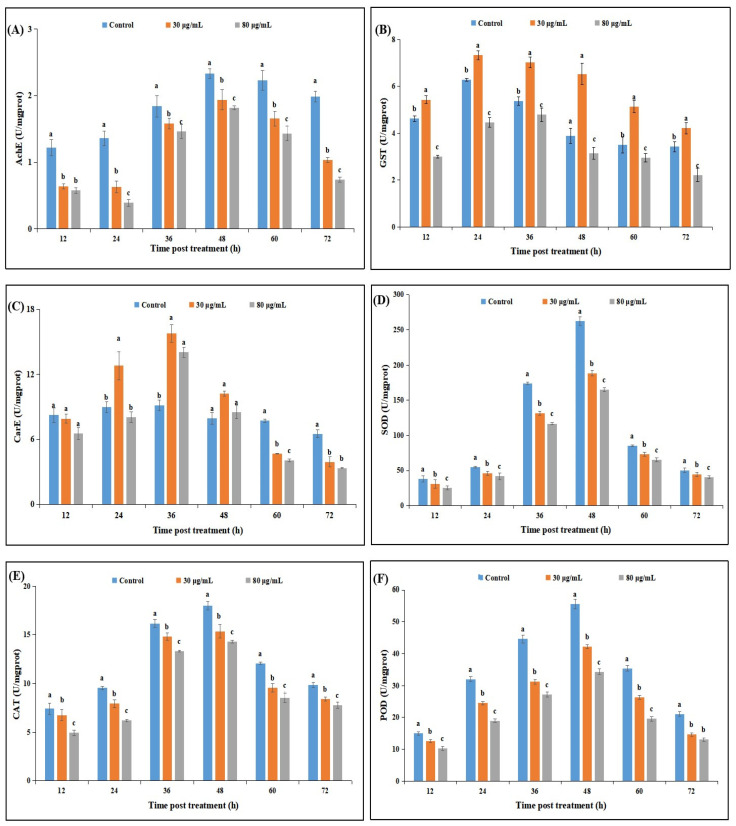
Effect of cyclosporin C on neurophysiological and antioxidant enzyme activities. (**A**) Acetylcholinesterase activity (AchE); (**B**) glutathione S-transferase (GST); (**C**) carboxylesterase (CarE); (**D**) superoxide dismutase (SOD); (**E**) catalase (CAT), and (**F**) peroxidase (POD). Bars represent standard error of means (based on three independent replicates). Bars with different letters represent significantly different means at different time intervals (Tukey’s, *p* < 0.05).

The acetylcholinesterase (AChE) activities of *P. xylostella* were significantly different among the cyclosporin C treatments and the control at different time intervals. The AChE activities of *P. xylostella* larvae treated with 30 μg/mL or 80 μg/mL cyclosporin C were significantly lower than those observed for the control. The lowest AChE activities were observed for the 80 μg/mL cyclosporin C treatment throughout the experimental period, whereas the highest (AChE) activities were observed for the control (Figure 5A).

The glutathione S-transferase (GST) activities of *P. xylostella* showed significant differences among the different cyclosporin C treatments and the control at different time intervals. The GST activities in response to different treatments showed an increasing trend until 36 h post-treatment, whereas a sharp decline in GST activities was observed at 36 h post-treatment until the end of the experimental periods. The lowest GST activities at different time intervals were observed for the 80 μg/mL cyclosporin C treatment, whereas the highest (AChE) activities at different time intervals were observed for the control treatments (Figure 5B).

The carboxylesterase (CarE) activities of *P. xylostella* were significantly different among the cyclosporin C treatments and the control at different time intervals. The CarE activities in response to different treatments showed an increasing trend until 36 h post-treatment, whereas a sharp decline in CarE activities was observed at 36 h post-treatment until the end of the experimental periods. The CarE activities of *P. xylostella* larvae treated with 30 μg/mL or 80 μg/mL cyclosporin C after 24, 36, and 48 h of treatment were significantly higher than the CarE activities observed for the control treatment. The lowest CarE activities throughout the experimental period (except 48 h post-treatment) were observed for the 80 μg/mL cyclosporin C treatment (Figure 5C).

The superoxide dismutase (SOD), catalase (CAT), and peroxidase (POD) activities of *P. xylostella* were significantly different among the cyclosporin C treatments and the control at different days post-treatment. The antioxidant enzyme activities in response to different treatments showed an increasing trend until 48 h post-treatment, followed by a sharp decline until the end of the experimental periods. The lowest antioxidant enzyme activities throughout the experimental period were observed for the 80 μg/mL cyclosporin C treatment, whereas the highest activities of antioxidant enzymes were observed for the control (Figure 5D–F).

## 3. Discussion

Data on the toxicity of microbial toxins (such as cyclosporins) against different insect pests are very limited to date [31]. This study demonstrated that cyclosporin C produced by entomopathogenic fungus *P. lilacinum* can induce lethal and sublethal effects against *P. xylostella*, which provides initial information about the potential of cyclosporin C for *P. xylostella* management.

Bioassay studies have revealed that different larval instars of *P. xylostella* were sensitive to different concentrations of cyclosporin C, showing a dose-dependent response. The median lethal concentrations (LC_50_) of cyclosporin C against first, second, third, and fourth instar larvae 72 h after application were 78.05, 60.42, 50.83, and 83.05 μg/mL, respectively. The transmission electron microscopy of the midgut of fourth instar *P. xylostella* larvae showed nuclei enlargement, folding of the nuclear membrane, and detachment of the microvilli from the midgut cells. Our results are similar to those of Weiser and Matha [12], who reported the toxicity of cyclosporin A against *Culex pipiens*, accompanied by histopathological changes to *C. pipiens* tissues (the formation of swollen mitochondria and vacuolization of the endoplasmic reticulum). Our results are also consistent with those of Vilcinskas et al. [5], who reported that an artificial diet supplemented with a sublethal concentration of cyclosporin A reduced the phagocytosis of plasmatocytes in *Galleria mellonella*.

To date, not many studies on microbial toxins have focused on their effects on the feeding rate, fecundity, and adult longevity of target insect pests, as these parameters can influence the ultimate population dynamics of insect hosts. Observations on antifeedant activity, larval growth rate, and adult emergence are very vital, as these variables are directly related to growth and reproduction [31]. Our results show a significant reduction in the amount of leaf consumed by *P. xylostella* per day, as well as an increase in the larval weight per day compared to the control after treatment with cyclosporin C. Different life history parameters, such as pupation rate (%), adult emergence (%), female fecundity, and female longevity, were also inhibited by different concentrations of cyclosporin C. These toxic properties of cyclosporin C can be related to the mode of action of cyclosporins. This group of fungal toxins has an ionophore-like mode of action that targets the Ca^+2^ level of invertebrate cells [32,33].

The biochemical responses by insect hosts to fungal are the least explained, and further elaboration of these responses is required to improve our knowledge of the toxicity of fungal cyclosporin C. Significant fluctuations in detoxifying and antioxidant enzyme activities of *P. xylostella* were observed post-cyclosporin C treatment. Insect immune systems respond to outer threats (pesticides or microorganisms) by regulating the enzyme activities of detoxifying enzymes, such as esterases and glutathione-S-transferase [34]. This study has revealed increased CarE and GSTs activities in *P. xylostella* larvae treated with cyclosporin C, confirming their role in cyclosporin C detoxification by *P. xylostella.* Our results are consistent with those of Luo and Zhang [35], who observed similar changes in the CarE and GSTs activities of *P. xylostella* treated with matrine. Acetylcholinesterase (AChE) terminates insect nerve impulses through acetylcholine breakdown at insects’ synapses [36]. Our results show an increase in AchE activities until 48 h post-cyclosporin C treatment, followed by a sharp decline. Antioxidant enzymes (SOD, CAT, and POD) are responsible for reducing damage caused by reactive oxygen species (ROS) [29]. We observed an increase in the SOD, CAT, and POD activities during the initial 48 h, followed by a reduction in enzyme activities post-cyclosporin C treatment. The reduced antioxidant enzyme activities might have resulted in the lower removal of the ROS and the denaturation of different bio-molecules, which led to higher insect mortality [37].

## 4. Conclusions

Our results show promising findings about the lethal and sublethal effects of cyclosporin C against *P. xylostella*, showing its potential for future development as a biocontrol agent of insects, such as the diamondback moth. These findings can serve as baseline information for the development of cyclosporin C as an insect control agent. However, further studies on the mass production, formulation, dose selection, and field application of cyclosporin C, as well as the ecological implications of its application (including potential biosafety studies on natural enemies and other living organism side effects) are still required for the successful development of cyclosporin C as a biopesticide.

## 5. Materials and Methods

### 5.1. Plutella xylostella and Cyclosporin C

The *Plutella xylostella* larvae used in this study were obtained from the stock cultures reared on *Brassica campestris* L. (Datou Aijiao cultivar; Guangzhou Changhe Seeds, Guangzhou China) at the Guangdong Key Laboratory of Biopesticide Innovation and Application, South China Agricultural University, Guangzhou [38]. Freshly hatched first instar larvae were used for anti-feedant assays, while fourth instar larvae were used for life history studies and enzyme assays.

Cyclosporin C was extracted and purified from strain XI-5 of *Purpureocillium lilacinum* (NCBI accession No. MW386435) following the method described by Burhan et al. [39]. The cyclosporin C was diluted to 500 μg/mL with dimethyl sulfoxide (DMSO) and stored at 4 °C until further use.

### 5.2. Toxicity of Cyclosporin C against P. xylostella

#### 5.2.1. Toxicity Assays

Different concentrations of cyclosporin C (25, 50, 100, 150, 200, and 300 μg/mL) were prepared from a stock solution through serial dilution using a 15% sucrose solution. The toxicity assays of cyclosporin C against different larval instars of *P. xylostella* were performed by oral route following the method by Ali et al. [40]. Briefly, cabbage leaf discs (10 cm^2^) were dipped for 30 s in different concentrations of cyclosporin C and air-dried (for 2 h) on paper. The leaf discs similarly treated with ddH_2_O served as the control. Freshly hatched first, second, third, and fourth instar *P. xylostella* larvae (30 individuals each) were transferred to *B. campestris* leaves from individual treatments (different concentrations and the control) via a camel hairbrush. *P. xylostella* larvae were fed on treated leaf discs for 48 h, followed by feeding on normal *B. campestris* leaf discs. The leaf discs were replaced every 48 h. The Petri dishes were incubated at 26 ± 1 °C and 55 ± 10% R.H. and 14:10 h (light/dark photoperiod). The leaf discs (treated and control) were replaced on a daily basis. The number of dead insects was counted on a daily basis. All the treatments and the control were repeated thrice with freshly hatched *P. xylostella* larvae.

#### 5.2.2. Transmission Electron Microscopic Examination of *P. xylostella* Midgut following Cyclosporin C Treatment

Fourth instar *P. xylostella* larvae were treated with two concentrations of cyclosporin C (30 and 80 μg/mL) and the control treatment (ddH_2_O) following the method described in Section 5.2.1. Changes in the appearance of the infected *P. xylostella* midgut were directly monitored at 4, 8, and 12 h post-treatment under a JEM1011 transmission electron microscope (Nikon Co. Ltd., Tokyo, Japan) following the method by Du et al. [36]. The treated larvae were sampled at 4, 8, and 12 h post-treatment and dissected under a stereo microscope (Stemi 508, ZEISS, Germany) to obtain midgut samples. The samples were fixed overnight in 2.5% glutaraldehyde +2% paraformaldehyde solution at 4 °C, followed by rinsing with PBS buffer (0.1 M). The samples were then stained overnight with 1% uranyl acetate at 4 °C, followed by dehydration with gradient concentrations of ethanol. The dehydrated tissues were embedded in silica gel blocks, and sections were cut using an automatic microwave tissue-processing instrument (EM AMW, Leica, Germany), and a cryo-ultramicrotome (EM UC7/FC7, Leica, Germany).

### 5.3. Antifeedant and Sublethal Toxicity of Cyclosporin C

#### 5.3.1. Antifeedant Activity Assays

For antifeedant assays, *B. campestris* leaf discs (10 cm^2^) were dipped in different concentrations of cyclosporin C (30 and 80 μg/mL) for 30 s, followed by air-drying (for 1 h) on Whatman Grade 1 filter paper. Leaf discs dipped in distilled water served as the control. Freshly hatched first instar *P. xylostella* larvae were transferred to the treatment and control leaf disks. The leaf disks were placed in Petri dishes lined with Whatman Grade 1 filter paper. The Petri dishes were incubated at 26 ± 1 °C and 55 ± 10% R.H. and 14:10 h (light/dark photoperiod). The *P. xylostella* larvae were fed on the treated leaf discs for 48 h, followed by feeding on normal *B. campestris* leaf discs. The leaf discs were replaced every 24 h. The feeding rate was defined as the change in weight of the *B. campestris* leaf discs after 24 h. The *P. xylostella* larvae from each treatment were weighed every 24 h on a weighing balance (Sartorius BCE Entriss II Analytical Balance) and the difference in the larval weight every 24 h was used as the change in larval weight per day. The effect of feeding on larval development was measured by changes in body weight during the observation period. All the treatments and the control were repeated thrice with freshly hatched *P. xylostella* larvae (four leaf discs with 20 larvae each).

#### 5.3.2. Sublethal Toxicity of Cyclosporin C

To determine the sublethal effect of cyclosporin C on pupation, adult emergence, adult longevity, and fecundity, a partial life cycle test was performed by feeding fourth instar *P. xylostella* larvae on *B. campestris* leaf discs (10 cm^2^) dipped in two concentrations of cyclosporin C (30 and 80 μg/mL) and the control treatment (ddH_2_O), as described in Section 5.3.1. The leaf disks were placed in Petri dishes lined with Whatman Grade 1 filter paper. The Petri dishes were incubated at 26 ± 1 °C and 55 ± 10% R.H. and 14:10 h (light/dark photoperiod). The *P. xylostella* larvae were fed on the treated leaf discs for 48 h followed by feeding on normal *B. campestris* leaf discs. The leaf discs were replaced every 48 h. The *P. xylostella* larvae were observed on a daily basis until adult emergence. All of the treatments and the control were repeated thrice. The pupation (%) and adult emergence (%) were calculated as follows:

Pupation (%) = (Number of successfully pupating larvae/Total number of larvae) × 100

Adult emergence (%) = (Number of adults emerged/Total number of pupae) × 100

The emerged adults (from each treatment group) were reared following the method of Ali et al. [40] to calculate the average number of eggs laid/day, and adult longevity.

### 5.4. Effect of Cyclosporin C on Neurophysiological and Antioxidant Enzyme Activities

Fourth instar *P. xylostella* larvae (50 individuals each) were treated with two concentrations of cyclosporin C (30 and 80 μg/mL) and the control treatment (ddH_2_O). Larvae from each treatment (5 individuals) were collected every 12 h post-treatment for enzyme assays. The larvae were homogenized in specific buffers for each enzyme at 4 °C following the method by Wu et al. [41]. Detailed information on the enzymatic assays can be seen in the Supplementary Materials. A total protein assay was performed with a total protein assay kit (Beyotime Biotech Inc., Shanghai, China), using bovine albumin serum as the standard [42].

Glutathione S-transferase (GSTs) activity assays were carried out according to Habig et al. [36]. Changes in absorbance were recorded spectrophotometrically (iMark microplate absorbance reader, Biorad, Feldkirchen, Germany) at 340 nm. A reduction in glutathione per minute per mg protein was used as a unit of enzyme activity.

Carboxylesterase (CarE) was quantified using 1-naphthyl acetate as a substrate [43]. The reaction mixture (0.45 mL 0.05 mol/L sodium phosphate buffer, 1.8 mL 0.00003 mol/L 1-naphthyl acetate, and 0.05 mL sample) was incubated in water at 30 °C for 15 min, followed by the addition of 0.9 mL 1% fast blue RR salt to terminate the reaction. The change in absorbance was measured at 600 nm. The enzyme activity was determined by the protein changes per unit over time.

The acetylcholinesterase (AChE) contents of *M. usitatus* from the different treatments were observed following the methods of Wu et al. [41] and Ellman et al. [44]. Changes in the absorbance were recorded spectrophotometrically (iMark microplate absorbance reader, Biorad, Feldkirchen, Germany) at 405 nm for 40 min. A unit of enzyme activity was defined as changes in the absorbance per minute per milligram of total protein.

The antioxidant enzyme (superoxide dismutase (SOD), catalase (CAT), and peroxidase (POD)) assays were performed with the respective enzyme assay kits provided by Nanjing Jiancheng Bioengineering Institute, Nanjing China. One unit of SOD was defined as the amount of enzyme required to suppress 50% of nitroblue tetrazolium at 560 nm [45]. One unit of CAT activity was defined as the amount of enzyme required to decompose 1 mmol H_2_O_2_/min [46]. One unit of POD activity was defined as changes in the absorbance per minute per milligram of protein [47].

### 5.5. Data Analysis

The data regarding mortality were arcsine-transformed, the means were compared using analysis of variance (ANOVA-2), and the significance of the means was compared using Tukey’s HSD test at a 5% level of significance. The data regarding the feeding rate, larval weight, percent of pupation, and percent of adult emergence were subjected to analysis of variance (ANOVA-1), and the significance of the means was compared using Tukey’s HSD test at a 5% level of significance. SAS 9.2 was used for all statistical analyses [48].

## Figures and Tables

**Table 1:** Sub-lethal effects of cyclosporin C on life history parameters of *Plutella xylostella*.

Life History Parameters	Control	30 μg/mL	80 μg/mL
Pupation (%)	96.66 ± 1.66 a	58.33 ± 1.40 b	14.44 ± 0.93 c
Adult emergence (%)	66.66 ± 2.33 a	23.33 ± 1.66 b	7.78 ± 1.11 c
Pre-oviposition period (h)	7.25 ± 0.66 c	9.33 ± 0.50 b	11.50 ± 0.33 a
Fecundity (eggs/female)	197 ± 4.48 a	137 ± 2.93 b	82 ± 2.34
Female longevity (d)	12 ± 0.33 a	9 ± 0.66 b	7 ± 0.66 c

Means (±standard error) in same row followed by different letters (a, b, c) are significantly different from each other (Tukey’s, *p* < 0.05).

## Data Availability

The raw data supporting the conclusion will be made available by the corresponding author on request.

## Supplementary Materials

The following are available online at https://www.mdpi.com/article/10.3390/toxins14080514/s1.
