# Supplementary material for: Lethal and Sublethal Toxicity Assessment of Cyclosporin C (a Fungal Toxin) against Plutella xylostella (L.)
Source: Toxins (Basel). 2022 Jul 28;14(8):514. doi: 10.3390/toxins14080514 (PMC9414777; doi:10.3390/toxins14080514)
Supplement: Supplementary file 1 [file toxins-14-00514-s001.zip › toxins-1810288-supplementary.pdf]

Supplementary Materials: Lethal and Sublethal Toxicity Assessment of Cyclosporin C (A Fungal Toxin) against *Plutella xylostella* (L.)

Jianhui Wu, Xiaochen Zhang, Muhammad Hamid Bashir and Shaukat Ali *

Pluttela xylostella Rearing

Pluttela xylostella larvae used in this study were obtained from the stock cultures reared on *Brassica campestris* L reared at Guangdong Key Laboratory of Biopesticide innovation and Application, South China Agricultural University, Guangzhou. *Brassica campestris* L (Datou Aijiao cultivar; Guangzhou Changhe Seeds, Guangzhou China) seeds grown in plastic pots using regular soil. Slow-release fertilizer (N: P: K=13:7:15, Shenzhen Batian ecotypic engineering Co., LTD. Xili Shenzhen China) was added as required to maintain normal plant growth. The pots were incubated in an artificial climate room at 26 °C and >95% R.H.

Effect of Cyclosporin C on Neurophysiological and Antioxidant Enzyme Activities

Fourth instar *P.xylostella* larvae (50 individuals each) were treated with two concentrations of Cyclosporin C (30 and 80 μg/mL) and control (ddH_2O). Larvae from each treatment (5 individuals) were collected every 12 h post treatment for enzyme assays. Larvae were were homogenized in 1.0 mL of specific buffers for each enzyme (0.05 mol/L Tris-HCL (pH 7.5) for GSTs assay; 0.02 mol/L PBS (pH 7.0) for AChE assay; 0.05 mol/L PBS (pH 7.8) for SOD assay; 0.15 mol/L PBS (pH 7.0) for CAT assay; and 0.2 mol/L PBS (pH=6.0) for POD assay) at 4°C followed by grinding with glass homogenizer on ice bath.The homogenates were centrifuged at 11,000 rpm for 10 min at 4 °C and the supernatant was taken as an enzyme source.

The glutathione S- transferase (GSTs) was conducted using the procedures developed by Habig (1981). Incubation was carried out at 25°C for 5 min in 0.1 M Na-phosphate buffer (pH 6.5) containing 1 mM glutathione, 1 mM DNCB, and 20 μl of the sample. The reaction was initiated by adding DNCB solution in acetone. Concentration of 5-(2,4-dinitrophenyl) glutathione produced during the reaction was measured spectrophotometrically at the wavelength of 340 nm. One unit of enzyme will conjugate 10.0 nmol of CDNB with reduced glutathione per minute.

Acetylcholinestrase (AChE) activity was assayed by following Ellman et al. (1961) with some slight modifications. The reaction mixture contained 50 μ L sample solution, 100 μ L 45 μ M 5-5-dithiobis-(2-nitrobenzoic acid), 100 μ L acetylthiocholine iodide and 90 μL sodium phosphate buffer. The change in absorbance at 405 nm was recorded for 40 min. One milliunit of AChE activity is the amount of enzyme that will generate 1.0 nmol of Choline per min.

Superoxide dismutase (SOD) activity was measured in cell free extracts by nitro blue tetrazolium (NBT) reduction (Beauchamp and Fridovich, 1971). The assay mixture contained 0.2ml 0.1M diamine diamine tetraacetic acid (EDTA) containing 0.3 mM sodium cyanide (0.2ml), 0.1 ml 1.5mM Nitroblue tetrazolium (NBT), 3ml 0.067M potassium phosphate buffer, pH 7.8 and a series of samples ranging from 0.1-10 micrograms protein in different tubes. The tubes were placed in a light box providing uniform light intensity. The tubes were incubated for 5-8 minutes to achieve a standard temperature. At zero time 0.05 ml 0.12 mM Riboflavin was added and all the tubes were incubated for 12 min and absorbance was read at 560nm after 1 min interval. One unit of SOD activity was defined as the amount of SOD required for inhibition of the reduction of NBT by 50% (A_{560}) and was expressed as units per mg protein (U/mg protein).

Catalase activity was assayed by the method described by Beers and Sizer (1952), in which the decomposition of H_2O_2 was analyzed spectrophotometrically at 240 nm. Reagent grade water (1.9 ml) and 0.059 M hydrogen peroxide (1.0 ml) were pipetted into the cuvette. The cuvette was incubated in spectrophotometer for 4-5 minutes to achieve temperature equilibration and to establish blank rate if any. After 5 min, 0.1 ml of sample was added and change in absorbance at 240 nm was observed for 2-3 minutes. Change in absorbance per minute was calculated from the initial (45 second) linear portion of curve. One unit of catalase activity was defined as the amount of enzyme that decomposes 1 mmol H_2O_2/min at an initial H_2O_2 concentration of 30 mM at pH 7.0 and 25 ºC and was expressed as units per mg protein (U/mg protein).

Peroxidase (POD) activity assay was performed by following Shannon et al. (1966). Briefly, 3.00 ml of reaction mixture having 2.1 mL H_2O, 0.32 mL 14 mM potassium phosphate buffer, 0.16 mL 0.027% (v/v) hydrogen peroxide,0.32 mL 0.5% (w/v) pyrogallol was incubated 20 °C for 10 minutes. The 0.1 mL of 14 mM potassium phosphate buffer and 0.1 mL of sample was added followed by mixing through inversion. Change in absorbance was measured spectrophotometrically at the wavelength of 420 nm. Enzyme activity was expressed as units per mg protein (U/mg protein).

The protein concentrations in the supernatants were determined by following the method of Bradford (1976) using bovine serum albumin as standard.
